# Persistent Hiccups—An Unusual Presentation of Bilateral Pheochromocytoma without Syndromic Association: A Case Report

**DOI:** 10.1155/2012/824030

**Published:** 2012-07-05

**Authors:** Nitin Aherrao, Nilesh Kumar, Indarajeet Singh Gambhir, Dhiraj Kishore, Suryakumar Singh, Abhinandan Mishra, Aravind Anand

**Affiliations:** ^1^Department of Medicine, Institute of Medical Sciences, Banaras Hindu University, Varanasi 221005, India; ^2^Department of Endocrinology and Metabolism, Institute of Medical Sciences, Banaras Hindu University, Varanasi 221005, India

## Abstract

Pheochromocytoma is a rare catecholamine-producing tumor arising from chromaffin tissue in the adrenal medulla, occurring in less than 0.2 percent of patients with hypertension. The mean age at diagnosis is about 40 years. Pheochromocytomas are commonly inherited as features of multiple endocrine neoplasia type 2 or several other pheochromocytoma-associated syndromes and have variable clinical presentation. Among the presenting symptoms, episodes of palpitations, headaches, and profuse sweating are typical and constitute a classic triad. We report a case of a 17-year-old male patient with rare bilateral pheochromocytoma presenting with persistent hiccups for 4 months and blurring of vision for 1 week, later followed by hypertensive crisis. There was neither family history of pheochromocytoma nor any classic symptoms. Patient was diagnosed with bilateral pheochromocytoma without any syndromic association. But still this patient needs to be followed for future development of medullary carcinoma of thyroid because it could be an initial presentation of MEN 2A/2B/VHL syndromes. Our paper highlights the importance of maintaining a high level of suspicion for persistent hiccups and careful clinical screening for hypertension even in absence of associated syndromes of pheochromocytoma and classical symptoms to achieve prompt diagnosis and to avoid improper management.

## 1. Introduction

Pheochromocytomas are rare catecholamine-producing tumours derived from the sympathetic or parasympathetic nervous system. They arise from chromaffin tissue in the adrenal medulla. These tumours may arise sporadically or be inherited as features of multiple endocrine neoplasia type 2 or several other pheochromocytoma associated syndromes. The clinical presentation is so variable that pheochromocytoma has been termed “*the great masquerader*.” They are estimated to occur in 2–8 of 1 million persons per year, and about 0.2% of hypertensive patients harbour a pheochromocytoma. The mean age at diagnosis is about 40 years, although the tumours can occur from early childhood until late in life. About 10% of pheochromocytomas are bilateral, however these percentages are higher in inherited syndromes. However, a pheochromocytoma can be asymptomatic for years, and some tumours grow to a considerable size before patients note symptoms. Here we report a case of bilateral pheochromocytoma presenting with persistent hiccups for 4 months and blurring of vision for 1 week. Patient presented without any classic symptoms of pheochromocytoma and did not have any syndromic association or significant family history.

## 2. Case Report 

A 17-year-old male presented to University Medical Centre with persistent hiccups for 4 months and blurring of vision (both eyes) for 1 week. On examination he was found to be hypertensive with blood pressure of 180/110 mm/Hg in both upper limbs with heart rate of 94 beats/min. Fundus examination showed bilateral papilledema with grade 4 hypertensive changes and rest physical examination was within normal limits. He was then evaluated for renovascular hypertension.

**Figure 1:**
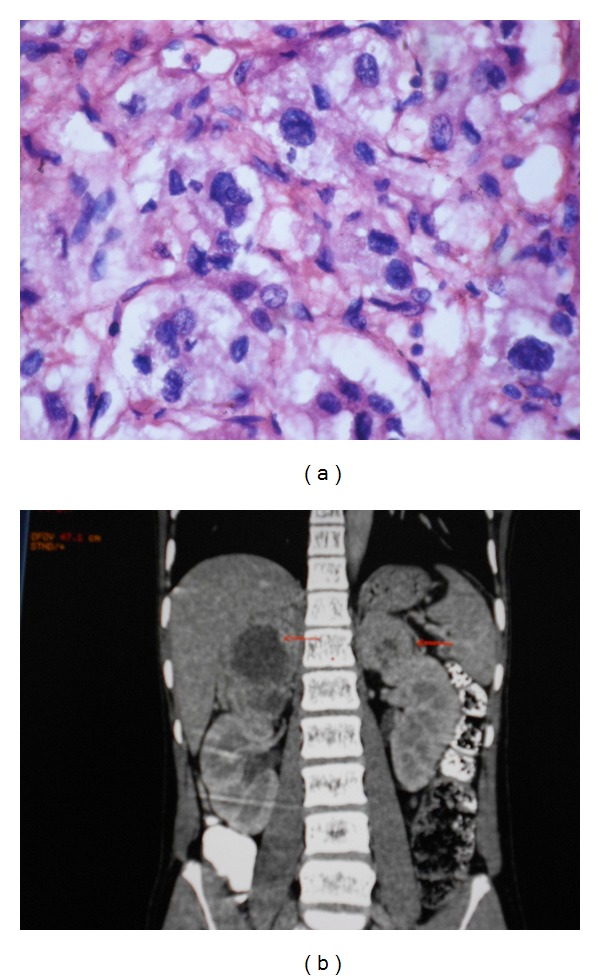
(a) Microscopic view of the resected mass. (b) CT scan Abdomen showing pheochromocytoma.

His routine investigations were as follows: Hb: 110 g/L, Creatinine: 55 micromol/L, Urea: 5 mmol/L, Na: 137 mmol/L, K: 3.8 mmol/L, Ca: 2.4 mmol/L, and Po_4 _: 1.1 mmol/L. His renal artery Color Doppler showed normal renal parenchyma and renal artery diameter but showed a bilateral suprarenal mass. Patient's CT abdomen was done that showed a relatively encapsulated, heterogeneously and moderately enhancing soft-tissue density mass (right—5.9 × 5.3 × 6.9 cm and left—3.8 × 3.9 × 4.6 cm) bilaterally in suprarenal area with internal necrotic and cystic areas (Figure 1(b)). 24 hr urinary metanephrines estimation was 450 mmol/mol creatinine (*N*—30–211 mmol/mol creatinine). Patient's 24 hr urinary vanillylmandelic acid estimation (VMA) was 137.86 micromol/day (*N*—2.02–77.91 micromol/day). In the meanwhile, patient was started initially on prazosin 5 mg twice daily which was then followed by addition of propranolol (40 mg) once daily resulted in blood pressure of 134/86 mm/Hg (supine). Patient was reevaluated but neither gave any significant family history nor had any signs suggestive of MEN 2A/2B/1, VHL syndromes, or neurofibromatosis. Patient did not show cushingoid habitus or signs of virilisation. Patient's calcitonin estimation was 2.0 ng/L (*N*—3–26 ng/L). MRI Brain did not show cerebellar or retinal hemangioblastoma. CT chest was within normal limit.

Patient was then shifted to the surgery department for surgical removal of the mass. Bilateral adrenlectomy was performed without any intraoperative complications. The mass was sent for histopathological examination. Postoperatively patient did not have complications, he got relieved off his hiccups, blood pressure was 130/70 mm/Hg off the antihypertensives, and he was supplemented with wysolone and fludrocortisone, awaiting histopathological examination. 

Histopathological examination of adrenal mass showed tumor cells arranged in mixed trabecular and nested pattern with marked nuclear pleomorphism and increased mitotic rate (Figure 1(a)). Areas of extensive necrosis and capsular invasion were noted. Immunohistochemistry of the tissue sample was positive for chromogranin. Post operative VMA was 35.3 micromol/day.

## 3. Discussion

Pheochromocytoma is a rare catecholamine-producing tumor arising from chromaffin tissue in the adrenal medulla but can also occur outside the adrenal in the abdomen, and are then called sympathetic paragangliomas [1]. They occur in less than 0.2 percent of patients with hypertension [2, 3]. Pheochromocytomas are often inherited as features of multiple endocrine neoplasia type 2 or several other pheochromocytoma-associated syndromes and have variable clinical presentation.

Bilateral pheochromocytoma of the adrenal glands is a rare entity. It has been mentioned previously in case reports but mostly those associated with syndromes like VHL, MEN2A/2B. Among the presenting symptoms, episodes of palpitations, headaches, and profuse sweating are typical and constitute a classic triad. The presence of all three symptoms in association with hypertension makes pheochromocytoma a likely diagnosis. Many patients may have atypical presentations which often delays diagnosis. However, a pheochromocytoma can be asymptomatic for years, and some tumors grow to a considerable size before patients note symptoms. Even with classical presentations, the diagnosis is often missed for a number of years unless the patient is evaluated in a centre experienced in this disease. Pheochromocytoma can have devastating consequences if not recognized and treated appropriately. 10% of pheochromocytomas are bilateral, extra-adrenal, and malignant. Pheochromocytomas in MEN2, VHL, and NF1 disorders usually are not the first clinical manifestation and are more likely to be benign and bilateral [4].

Our patient presented mainly with persistent hiccups for a long duration which was then followed by hypertensive crisis. Patient did not have any classic symptoms of pheochromocytoma. Patient had bilateral pheochromocytoma without any syndromic association. The dominant sign is hypertension. Classically, patients have episodic hypertension, but sustained hypertension is also common. Catecholamine crises can lead to heart failure, pulmonary edema, arrhythmias, and intracranial hemorrhage. During episodes of hormone release, which can occur at very divergent intervals, patients are anxious and pale, and they experience tachycardia and palpitations. Although pheochromocytomas rarely metastasize, they can be lethal because of intractable hypertension or anesthesia-induced hypertensive crises. Pheochromocytomas occur in 50% of individuals with MEN 2B; about half are multiple and often bilateral. Individuals with undiagnosed pheochromocytoma may die from a cardiovascular hypertensive crisis perioperatively [5].

Brief episodes of hiccups that self-terminate or that respond to simple maneuvers need no investigation or follow-up care. In contrast, persistent and intractable hiccups are a more serious phenomenon and often a diagnostic dilemma. These attacks have been frequently associated with an underlying pathological process and may induce significant morbidity and even death. The focus of the history, examination, and investigation is to identify these causes and effects. A full systemic inquiry, surgical history, and comprehensive drug history may reveal one of the many causes. Among several causes of hiccups are psychogenic causes, vagus nerve irritation, diaphragmatic irritation, drugs, and arrhythmia-induced syncope [6] procedure/anaesthesia related [7, 8].

Previously hiccups have not been described in the literature of pheochromocytoma which was unusual in this case. Hiccups might have been because of bilateral phrenic nerve irritation. So, practitioners must maintain high level of suspicion while evaluating hiccups because it could be an unusual presentation of pheochromocytoma. The diagnosis of pheochromocytoma provides a potentially correctable cause of hypertension, and their removal can prevent hypertensive crises that can be lethal. The clinical presentation is variable, ranging from an adrenal incidentaloma to a patient in hypertensive crisis with associated cerebrovascular or cardiac complications.

Patient, diagnosed with bilateral pheochromocytoma at a young age, neither gave any significant family history nor had any signs suggestive of MEN 2A/2B/1, VHL syndromes, or neurofibromatosis. But, this patient needs to be followed for future development of medullary carcinoma of thyroid because it could be an initial presentation of MEN 2A/2B/VHL syndromes.

## 4. Conclusion

The case we report here is uncommon because the patient presented with persistent hiccups. The report also emphasizes following clinically important issues, although extremely rare, bilateral pheochromocytoma can exist without syndromic association or significant family history can present at a young age as in this patient in absence of classical symptoms. Our intention is to alert Physicians to maintain a high level of suspicion for persistent hiccups and careful clinical screening for hypertension even in absence of classical symptoms or associated syndromes of pheochromocytoma to achieve prompt diagnosis and to avoid improper management.

## References

[1] Lenders JWM, Eisenhofer G, Mannelli M, Pacak K (2005). Phaeochromocytoma. *The Lancet*.

[2] Pacak K, Linehan WM, Eisenhofer G, Walther MM, Goldstein DS (2001). Recent advances in genetics, diagnosis, localization, and treatment of pheochromocytoma. *Annals of Internal Medicine*.

[3] Stein PP, Black HR (1991). A simplified diagnostic approach to pheochromocytoma. A review of the literature and report of one institution’s experience. *Medicine*.

[4] Neumann HPH, Berger DP, Sigmund G (1993). Pheochromocytomas, multiple endocrine neoplasia type 2, and von Hippel- Lindau disease. *The New England Journal of Medicine*.

[5] Erlic Z, Rybicki L, Peczkowska M (2009). Clinical predictors and algorithm for the genetic diagnosis of pheochromocytoma patients. *Clinical Cancer Research*.

[6] Suh WM, Krishnan SC (2009). Violent hiccups: an infrequent cause of bradyarrhythmias. *Western Journal of Emergency Medicine*.

[7] Salanitri S, Gonçalves AJ, Helene A, Lopes FHJ (2009). Surgical complications in hair transplantation: a series of 533 procedures. *Aesthetic Surgery Journal*.

[8] Doshi H, Vaidyalingam R, Buchan K (2008). Atrial pacing wires: an uncommon cause of postoperative hiccups. *British Journal of Hospital Medicine*.

